# Chionanthus in Jaundice

**Published:** 1896-06

**Authors:** H. C. Morrow

**Affiliations:** Austin, Texas


### CHIONANTHUS IN JAUNDICE.

H. C. MORROW, M. D., AUSTIN, TEXAS.

Mrs. A. had suffered several days with a very severe headache before sending for me.

Pains shoot like lightning from temples to eyes—right temple worse than the left. Moving of the eyeballs causes aggravation. Pressure relieves. Right hand and arm get cold. Feels sore all over. Scalp is sore. Lies very quiet. Is afraid to move because motion aggravates all the pains. Moistens lips with tongue. Drinks frequently but not much at a time. Throbbing in temples. Sighs and moans and is in very great distress. Motion and jar aggravate headache. Face and teeth sore. All complaints worse on right side. Right hand warm, left hand cold.

Photophobia — eyeballs very sore. It hurts to move them and aggravates headache, also chill every day about 8 P. M., which soon anticipated to 5 P. M.

Thirst before and during chill; aching all over. Felt cold in stomach. Felt subjectively cold, but objectively warm. Thirst for small quantities of water frequently during the fever, but it is vomited in a short time.

Wants cold drinks; craves buttermilk. Headache better lying on painful side. Hot to touch during chill.

Covered up during chill, but uncovers during fever. Wants to be uncovered during sweat. Thirst during sweat.

Burning thirst during fever for very cold drinks, which are vomited as soon as they become warm in the stomach.

Great restlessness and distress, has to move occasionally although motion is intensely painful and does not ameliorate. Sore all over—sore to touch when handled. Is so sore she cannot move herself, has to be helped.

Back and legs ache during chill and fever. Cold inside, hot outside during chill. Very profuse sweat after fever passes off. Burning like fire the whole length of œsophagus. Acids disagree. Urine burns when it passes. Very severe boring, pressing pains in stomach.

Pains in stomach worse from motion, turning, gaping, belching, breathing, lying on back, eating and drinking.

Stomach and whole length of œsophagus burns like fire. Great restlessness and anguish.

After she had been sick several days and no amelioration of her sufferings from Arsenicum, Arnica or Bryonia, she became jaundiced. Skin yellow as saffron and sclerotics deep greenish-yellow, but no amelioration of her sufferings. She was five months pregnant, but strange to say with all her vomiting and retching and agony she had no intimation of a miscarriage.

After the appearance of the jaundice I put her on a Chionanthus$^{30}$ (my own potency), and from the very first there was improvement and in two days she was apparently as well as she had ever been in her life.

She went to full term and was delivered of a strong, healthy child.

I did not test the urine, but always have been sorry I did not. There may have been some albumen in the urine, but of course this is conjecture.

Chionanthus is a remarkable remedy in disorders of the liver, especially where the appearance of the skin and eyes show that the liver is not secreting the bile.

An uncomplicated case of jaundice I have never failed to cure in a few days with this remedy.

I cured with Chionanthus, a year ago, an old German who had had jaundice of several weeks standing. In less than a week all traces of jaundice were gone, and he has been in fine health ever since.

Not only that, but severe attacks of gall-stone colic disappeared with the jaundice. These he had been having at frequent intervals for two years.

I quote a few symptoms from its proving to show its intense action on the pneumogastric nerve and on the muscular system. The following are the symptoms of

